# Differences in the Plasma Proteome of Patients with Hypothyroidism before and after Thyroid Hormone Replacement: A Proteomic Analysis

**DOI:** 10.3390/ijms19010088

**Published:** 2018-01-01

**Authors:** Assim A. Alfadda, Hicham Benabdelkamel, Afshan Masood, Anwar A. Jammah, Aishah A. Ekhzaimy

**Affiliations:** 1 Obesity Research Center, College of Medicine, King Saud University, P.O. Box 2925 (98), Riyadh 11461, Saudi Arabia; hbenabdelkamel@ksu.edu.sa (H.B.); afsmasood@ksu.edu.sa (A.M.); 2 Department of Medicine, College of Medicine, King Saud University, P.O. Box 2925 (38), Riyadh 11461, Saudi Arabia; ajammah@ksu.edu.sa (A.A.J.); aishahekhzaimy@hotmail.com (A.A.E.)

**Keywords:** hypothyroidism, l-thyroxine, proteomics, inflammation, acute phase proteins

## Abstract

Thyroid hormone is a potent stimulator of metabolism, playing a critical role in regulating energy expenditure and in key physiological mechanisms, such as growth and development. Although administration of thyroid hormone in the form of levo thyroxine (l-thyroxine) has been used to treat hypothyroidism for many years, the precise molecular basis of its physiological actions remains uncertain. Our objective was to define the changes in circulating protein levels that characterize alterations in thyroid hormone status. To do this, an integrated untargeted proteomic approach with network analysis was used. This study included 10 age-matched subjects with newly diagnosed overt hypothyroidism. Blood was collected from subjects at baseline and at intervals post-treatment with l-thyroxine until they reached to euthyroid levels. Plasma protein levels were compared by two-dimensional difference in gel electrophoresis (2D-DIGE) pre- and post-treatment. Twenty differentially expressed protein spots were detected. Thirteen were identified, and were found to be unique protein sequences by MALDI-TOF mass spectrometry. Ten proteins were more abundant in the hypothyroid vs. euthyroid state: complement C2, serotransferrin, complement C3, Ig κ chain C region, α-1-antichymotrypsin, complement C4-A, haptoglobin, fibrinogen α chain, apolipoprotein A-I, and Ig α-1 chain C region. Three proteins were decreased in abundance in the hypothyroid vs. euthyroid state: complement factor H, paraneoplastic antigen-like protein 6A, and α-2-macroglobulin. The differentially abundant proteins were investigated by Ingenuity Pathway Analysis (IPA) to reveal their associations with known biological functions. Their connectivity map included interleukin-6 (IL-6) and tumour necrosis factor α (TNF-α) as central nodes and the pathway identified with the highest score was involved in neurological disease, psychological disorders, and cellular movement. The comparison of the plasma proteome between the hypothyroid vs euthyroid states revealed differences in the abundance of proteins involved in regulating the acute phase response.

## 1. Introduction

Thyroid hormones, 3,5,3′,5′-l-tetraiodothyronine (thyroxine [T4]) and 3,5,3′-l-triiodothyronine (T3), are known to affect and act on every cell and organ of the human body. Their actions are mediated through both genomic and non-genomic effects. Briefly, they are known to promote normal growth and development and to regulate different homeostatic functions, including energy expenditure and heat production through their genomic actions [1] and regulate important cellular functions such as differentiation, proliferation, migration, and cell survival in all cell types, including cardiomyocytes, hepatocytes, and immune cells by the non-genomic actions [2]. Hypothyroidism is a clinical syndrome resulting from thyroid hormone deficiency, and is characterized by a generalized slowing down of metabolic processes. Patients with hypothyroidism often suffer from fatigability, cold intolerance, and weight gain. Besides the known biochemical changes that is seen in patients with overt hypothyroidism, such as elevated serum levels of total cholesterol, low density lipoprotein cholesterol (LDL-C), and apolipoprotein B [3], alterations in the immune response are also noted. These are characterized by decreased antibody production, cell migration, and lymphocyte proliferation, along with increased levels of reactive oxygen species, and the expression of pro-inflammatory molecules, such as macrophage inflammatory protein-1α and interleukin-1β [4]. Although several studies have confirmed a relationship between thyroid hormones and immunity, the exact mechanisms of this relationship still remains uncertain [4,5,6]. 

There has been much progress in our understanding of the transcriptional regulation of thyroid hormones and their genomic actions on the different target genes; however, little is still known about the relationship between thyroid hormone levels and the plasma proteome profile. An untargeted proteomics approach, based on two dimensional difference in gel electrophoresis (2D-DIGE) mass spectrometry, serves as a powerful tool in identifying these changes and was used earlier in studies that were primarily focused on thyroid cancers or nodules [7,8,9]. Understanding the changes that occur in the plasma proteome during a hypothyroid state and the changes that may occur upon achieving normal circulating thyroid hormone levels will help in further exploring their metabolic effects. To our knowledge, this is the first report on plasma proteome changes after thyroid hormone replacement in patients with hypothyroidism. Silvestri et al. used a proteomic approach to study protein expression changes in rat liver following the administration of T3, and identified fourteen differentially expressed proteins that were involved in several biochemical and metabolic pathways in the liver [10]. Engelmann et al. experimentally induced thyrotoxicosis in healthy volunteers to explore the effects of l-thyroxine excess on the plasma proteome with a main focus on coagulation markers [11]. Therefore, the aim of our study was to evaluate changes in the plasma proteome in patients with hypothyroidism after replacement treatment with appropriate doses of l-thyroxine using the 2D-DIGE mass spectrometry approach [12]. Exploring the plasma proteome changes upon the transition from a hypothyroid to euthyroid state will help in understanding the thyroxine-mediated changes taking place in circulating proteins.

## 2. Results

### 2.1. Biochemical Parameters of the Study Subjects

The laboratory characteristics of the participants are summarized in Table 1. As expected, statistically significant changes (*p* value < 0.001) in biochemical profile were noted for FT4 and TSH values after the treatment with l-thyroxine. No differences were noted in any of the other biochemical parameters. Multiplex analysis for the serum cytokines showed that the mean levels for TNF α and IL 6 were found to be higher in the hypothyroid group when compared to the euthyroid group, but without any statistical significance. 

### 2.2. 2D-DIGE Analysis and Identification of Differentially Abundant Proteins

**Figure 1:**
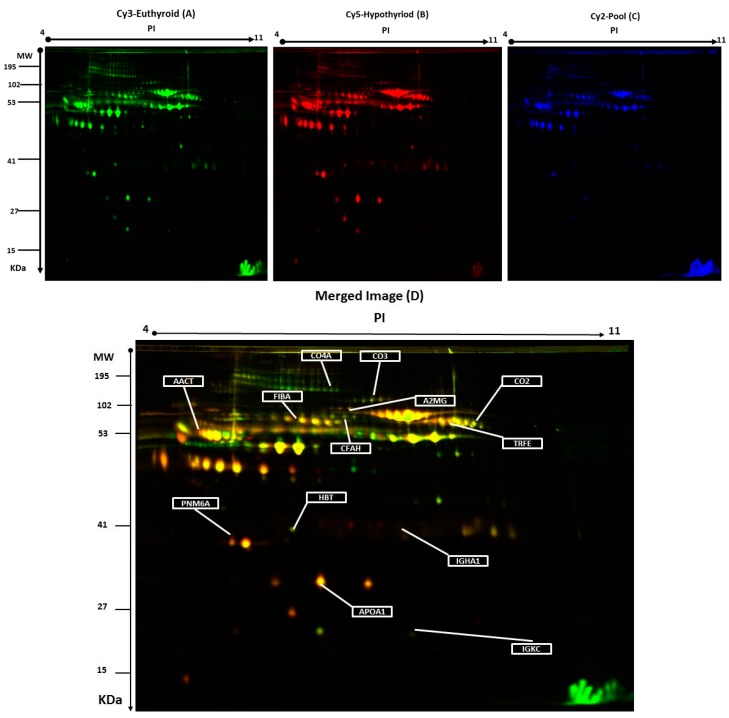
Representative two-dimensional difference in gel electrophoresis (2D-DIGE) images of the plasma proteins. Protein samples from hypothyroid and euthyroid groups were labeled with Cy3 or Cy5, shown together with a pooled internal standard sample labeled with Cy2, and separated by 2D-DIGE. Gels were scanned and a set of images: (**A**) Cy3 (**B**) Cy5 and (**C**) Cy2 were obtained from each gel. An overlay of all three images (**D**). The arrows indicate differentially regulated protein spots that were determined by image analysis and identified by MALDI-MS.

Plasma protein levels were compared by two-dimensional difference in gel electrophoresis (2D-DIGE) between the hypothyroid vs. euthyroid states. Figure 1 shows a representative 2D-DIGE gel from the set of pairwise comparisons that were made between the two groups. 

The arrows indicate the differentially abundant spots that were either decreased or increased between the groups. The spot patterns were reproducible across the 10 gels; therefore, alignment and further analysis were possible. Cy2-labeling (internal standard) was included to permit normalization across the complete set of gels and quantitative differential analysis of the protein levels. Significant changes in protein abundance were determined by ANOVA (i.e., *p*-value ≤ 0.05 and fold-change ≥ 1.5). Progenesis statistical software analysis detected a total of 20 protein spots, showing a significant increase or decrease in expression between the hypothyroid and euthyroid state. In some cases, variants of the same protein were found at several locations on the gel. For 13 of the 20 spots, peptide mass fingerprints (PMF) were successful, and were found to be unique protein sequences by MALDI-TOF mass spectrometry and were matched to entries in the SWISS-PROT database by Mascot with high confidence. Ten proteins were more abundant in the hypothyroid vs. euthyroid state: complement C2 (spot #394), serotransferrin (spot #384), complement C3 (spot #509), Ig κ chain C region (spot #1120), α-1-antichymotrypsin (spot #756), complement C4-A (spot #625), haptoglobin (spot #1019), fibrinogen α chain (spot #634), apolipoprotein A-I (spot #1150), and Ig α-1 chain C region (spot #713). Three proteins were decreased in abundance in the hypothyroid vs. euthyroid state: complement factor H (spot #270), paraneoplastic antigen-like protein 6A (spot #1110), and α-2-macroglobulin (spot #774). A list of the 13 protein spots identified as differentially abundant between the hypothyroid vs. euthyroid state are listed in Table 2 and Figure S2. Additional DIGE analysis was carried out using the same criteria as mentioned above, in order to determine for any variation between the genders and no significant changes in the spots was observed. 

### 2.3. Confirmation of Changes in Selected Proteins by Immunoblotting

**Figure 2:**
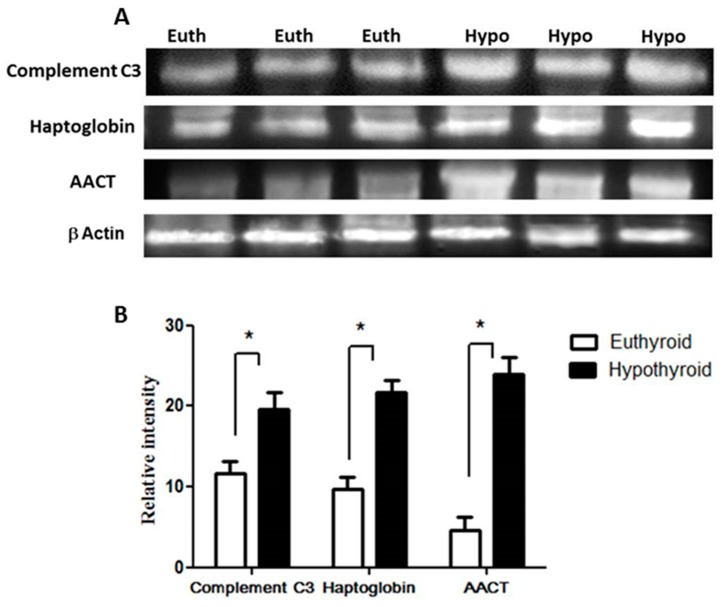
Confirmation of the proteomic data using immunoblot analysis of selected proteins, identified by 2-DE analysis. (**A**) Results obtained by immunoblotting were similar to the results obtained by 2D-DIGE; (**B**) Graphical representation of the relative intensity values of normalized protein bands between the hypothyroid and euthyroid states. The data are reported as histograms of the mean ± SEM and statistical significance at *p* ≤ 0.05 (indicated by *).

Key proteins that were found to be differentially abundant between the hypothyroid and the euthyroid states were confirmed by immunoblot analysis (Figure 2). The proteins selected for confirmation were as follows: complement C3, haptoglobin, and α-1-antichymotrypsin. The immunoblots confirmed the differential expression of these three plasma proteins between the two states. Immunoblot data were normalized to the housekeeping protein β-actin.

### 2.4. Protein–Protein Interaction Networks

**Figure 3:**
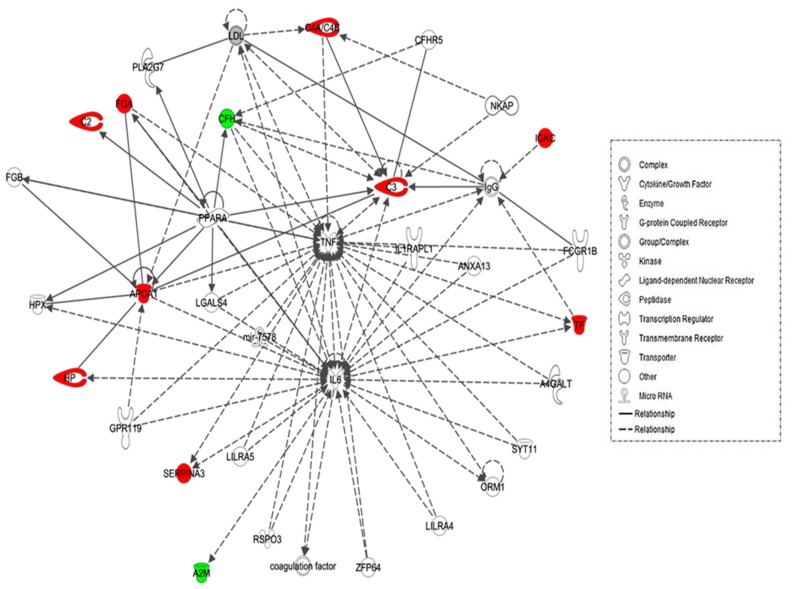
Schematic representation of the most significant Ingenuity pathway analysis (IPA) networks from differentially regulated proteins between the hypothyroid and euthyroid states: IPA analysis found the functional interaction networks pathway with the highest score of 28 related to “neurological disease, immunological disease, metabolic disease” showing tumour necrosis factor (TNF) and IL-6 as central nodes that were dysregulated in hypothyroidism. Nodes in green and red correspond to down and up regulated proteins respectively. Colourless nodes were proposed by IPA and suggest potential targets functionally coordinated with the differential proteins. Solid lines indicate direct molecular interactions and dashed lines represent indirect relationships.

The biological roles of the differentially abundant proteins identified between the hypothyroid and euthyroid states were investigated using IPA software. This software computes a score based on the best fit of the input data set of proteins and a list of biological functions stored in its knowledge base to generate a network pathway of significance. The generated network is preferentially enriched for proteins with specific and extensive interactions, the interacting proteins being represented as nodes and their biological relationship as a line. In our data, two interaction networks were identified for the proteins exhibiting differential expression (Table S2). The highest scoring network incorporated 11 out of the 13 focus molecules (Figure 3). The most interesting canonical pathways included acute phase response signaling (ratio = 0.0551, *p* = 2.35 × 10^−17^) and complement system (ratio = 0.105, *p* = 2.33 × 10^−5^). A strong association between the dataset and the respective pathway was indicated by the small *p*-value. Details of the canonical pathways that were identified in this study are summarized in Table S2.

### 2.5. Classification of Key Proteins Based on Function

**Figure 4:**
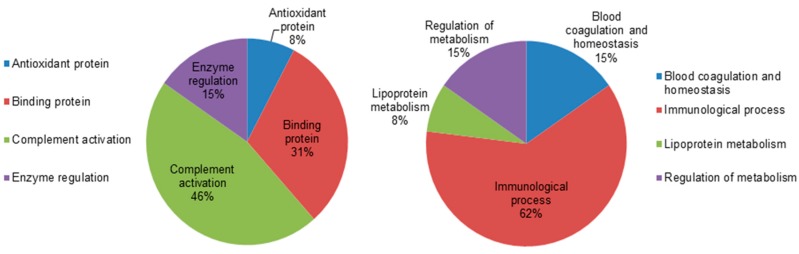
Comparative depiction of the differentially abundant identified proteins categorized into groups according to their function based on the gene ontology (GO) terms using UniprotKB. The representative pie diagram shows the (%) of involvement for different functional categories of the proteins between the hypothyroid and euthyroid states.

To gain a better understanding of the functional role of the different proteins that were identified in the dataset, the UNIPROT system and gene ontology GO were used to assign functions to the differentially expressed proteins between the hypothyroid and euthyroid states. The proteins identified were found to function mainly as enzymes, proteins involved in enzyme regulation, antioxidant proteins, binding proteins, and complement activation proteins. Figure 4 depicts graphically the percentage of functional involvement of these proteins in the study. 

### 2.6. Principal Component Analyses (PCA) Analyses

**Figure 5:**
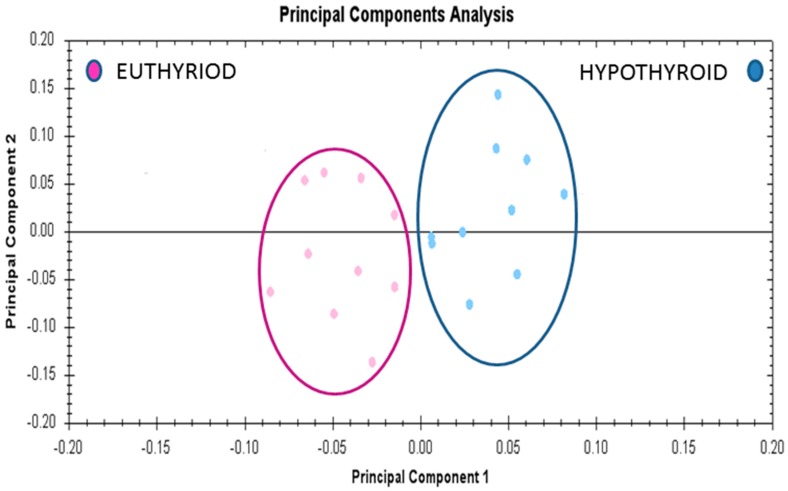
Principle component analysis of the two groups depending on the treatment state (hypothyroid vs. euthyroid state). Ellipses have been drawn to illustrate their clustering. This analysis is based on the 20 spot features exhibiting statistically significant (ANOVA *p* < 0.05) changes in abundance that were present on all 20 gel images (PC1 = 45.7, PC2 = 13.2).

Multivariate analyses of the protein abundance data were performed using Progenesis Same Spots software. The gel images were grouped such that 10 samples from the hypothyroid and 10 from the euthyroid state formed two groups. The data were filtered so that only the 20 spot features exhibiting statistically significant (ANOVA *p* ≤ 0.05) changes in abundance, present on all gel images, and identified by MS, were considered. The analyses revealed that the two groups clustered distinctly from one another based on treatment (Figure 5).

## 3. Discussion

The etiopathogenesis of thyroid disease is predominantly attributed to a dysfunction of the autoimmune system, called autoimmune thyroid disease, which encompasses a broad spectrum of thyroid disorders. Primary hypothyroidism is considered to form one end of this spectrum as Hashimoto’s thyroiditis and is clinically characterized by decreased free thyroxine (T4) and increased TSH circulating levels. Studies have examined additional plasma markers for hypothyroidism by studying the effect of physiological decreases in thyroid hormones (TH) on plasma proteins by utilizing either animal models [13], cell lines [14], and more recently in humans with induced hypothyroidism by administering l-thyroxine [15]. It is well-known that TH regulates plasma protein synthesis and secretion through its actions on target genes and their response elements [16]. Although the metabolic actions of TH are well-established, an overall circulatory plasma proteome and the changes therein have not been mapped in patients with hypothyroidism before and after l-thyroxine treatment. A complex and reciprocal relationship is also known to exist between the endocrine and immune system, wherein TH play a role in maintaining lymphocyte subpopulations and positively mediating the inflammatory response by increasing inflammatory markers [17]. This relationship has been explored earlier by a targeted approach using specific antibodies and an untargeted approach through proteomics and metabolomics applications. In this study, we tried to close this gap through evaluating the differences in the plasma proteome of individuals with hypothyroidism and the changes that occur upon their transition from a hypothyroid to a euthyroid state by administering l-thyroxine.

The thirteen proteins identified through our exploratory proteomics study were identified as acute phase proteins (APPs), which are part of the non-specific and complex acute phase response (APR) that is known to increase due to various stressors [18]. Classically, during this response, the acute phase proteins are considered to represent the positive phase when increased and negative phase when decreased. Twelve of the identified proteins in our dataset belonged to this classical positive phase and one of them to the negative phase, although their levels in our study were different from previous findings and did not correspond to the classical presentation. 

### 3.1. Proteins with Increased Abundance in the Hypothyroid vs. Euthyroid State 

In the present study, we identified three proteins that were associated with the complement system; namely C2, C3, and C4a, with significantly higher abundance in the hypothyroid state. Complement proteins, known to be synthesized primarily by B lymphocytes, are also synthesized by the thyroid cells i.e., the thyrocytes [19]. Thyrocyte metabolic function is impaired by the activation of complement takes on either the classical, lectin, or alternative pathways that converge on complement C3, which is regulated by thyroid hormones [20]. Our findings are similar to those of Yu and Jafarzadeh et al., who observed increased C3 levels in hypothyroidism [20,21], and with Parkes, who documented their increase in postpartum thyroiditis [22]. We have additionally identified C2 and C4a, a short-lived fragment of C4, in our data, which indicates a higher probability for involvement of the classical and lectin complement pathways in thyroid disease that are activated through antibody-independent pathways. 

The complement cascade is also at the interface of coagulation and inflammation pathways and acts as the mediator of inflammatory tissue damage in Hashimotos thyroiditis. Elevated levels of C2, C3, and C4a are also likely due to increased hepatic synthesis in response to activation of APR and cytokines, namely IL-1β, IL-6, or TNF-α which are increased in the hypothyroid state [23]. The C4a component additionally has been associated with other autoimmune inflammatory and infectious diseases, as well as with neurological disorders [24]. This interaction of thyrocytes with the immune system occurs through the expression of immunologically active molecules and a sub-lethal activation of complement cascade, which further exacerbates the autoimmune process, increasing cytokine production and the risk for destructive thyroiditis.

Another interesting finding from our comparison between the hypothyroid and the euthyroid states was a significant increase in the abundance of spots relating to Apo A1 [25]. TH’s are known to bind to lipoproteins, mainly the high-density lipoprotein, of which ApoA1 forms the major fraction [26]. Previous studies have been inconclusive regarding levels of ApoA1, as some have shown an increase while others have shown that it is not affected by TH status [27,28]. Increased ApoA1 levels were shown to increase the activity of type 1 deiodinase enzyme, which in turn regulates ApoA1 synthesis [29] and was shown to preferentially deiodinate reverse T3, sulfated iodothyronines, and participates in the biosynthesis of thyronamines that are known to antagonize TH actions [30]. 

Aside from ApoA1, we also identified serotransferrin, a metal cofactor transport protein that is known for its functional role in transporting iron, which is also required for activity of the deiodinase enzyme [31]. We found an increase in the abundance of protein spots relating to serotransferrin in the hypothyroid state, contrary to it being considered as a classical negative-phase APP. Lin et al., in their study in hepatoma cell lines (HepG2-TRa1), showed a direct induction of serotransferrin by T3. Serotransferrin has also been found to enhance thyroid response elements and is involved in the action of thyroid hormones during myelinogenesis [32] 

Thyroid hormones are known to affect the hemostatic system at various levels. Previous studies have shown that hypothyroidism is associated with both hypocoagulable and hypercoagulable states [33]. In our study, we identified that spots relating to fibrinogen and haptoglobin were significantly increased in the hypothyroid state. Chadarevian et al. demonstrated a negative and independent relationship between fibrinogen and FT4. It has been hypothesized that fibrinogen acts as a link between prothrombotic and proinflammatory states and promotes a more coagulable state, as seen in patients with normal-low FT4 levels compared to those with normal-high FT4 levels [34]. It is thus plausible based on our findings to postulate that with hypothyroidism, the delicate balance between coagulation and fibrinolysis is tilted towards a more procoagulable state. Functionally, an elevation in the plasma fibrinogen levels in hypothyroid patients have been associated with an increased risk of cardiovascular events. Physiologically, haptoglobin is well-known as a hemoglobin-binding protein with antioxidant and anti-inflammatory activity, and is capable of stimulating angiogenesis [35,36]. Haptoglobin expression levels are known to increase with inflammation and infection and these may involve immunogenic mechanisms that are aimed at reducing oxidative stress within the thyroid follicles. Our findings are in line with those of Lin et al. who showed increased haptoglobin levels upon hypothyroidism in hepatoma cell lines [16]. 

Spots relating to α1-antichymotrypsin, a serine protease inhibitor that was involved in regulating proteolytic enzymes critical for maintaining homeostasis, extracellular matrix remodeling, and conversion of prohormone to the active form, were significantly increased in our study. The low-grade inflammation seen in the hypothyroid state through induction of the TNF-α and NF-κB pathways via cytokines is further aggravated by antichymotrypsin, which induces further activation of TNF-α and NF-κB in an autocrine manner [37,38]. To date, antichymotrypsin has only been documented by Lai et al. in tumor cells from papillary carcinomas of the thyroid gland with a probable role in cancer progression [39]. The presence of low-grade inflammation is further signified by the increased number of spots relating to immunoglobulin κ chain C and α 1 chain C, even after using the albumin and IgG removal kit (GE Healthcare, Buckinghamshire, UK), which may suggest a true abnormality in the κ-free light chain in the plasma of patients with hypothyroid. Increased IgG levels were shown by Noh et al. to inhibit thyroid hormone synthesis in cultured slices of normal human thyroid tissue, leading to hypothyroidism [40]. Our findings are consistent with those of Yamauchi et al., who also observed an increase in the levels of IgG in 95 patients with Hashimoto’s thyroiditis [41]. 

### 3.2. Proteins Found with Decreased Abundance in Hypothyroid vs. Euthyroid State

We found a reduced abundance of α2-macroglobulin, a classical positive APP and antiproteinase, in the hypothyroid state. A2M inhibits all four classes of proteinases; namely, trypsin, thrombin, and collagenase, and is also a known cytokine transporter. This finding is consistent with those of Lin et al., who also observed decreased α2-macroglobulin levels in hypothyroid individuals [42]. Other proteomic studies also identified α2-macroglobulin as a potential biomarker in numerous diseases [43]. 

Complement Factor H is a relatively abundant plasma protein that is essential for maintaining complement homeostasis through regulating the alternative pathway. We observed a significant decrease in the abundance of this protein in the hypothyroid state when compared to levels in the euthyroid state. A decrease in factor H concentration may again indicate decreased regulatory control of the alternate pathway, which has been implicated in various diseases [44] and conditions of cellular stress, such as hypoxia [45].

Thyroid hormones play an important role in the neurological development of the brain and neurons by acting on migration and differentiation of neural cells, synaptogenesis, and myelination. A group of novel neuronal proteins, grouped as paraneoplastic neuronal antigens expressed by neural cells, are known to be involved in apoptosis and regulating mRNA and biological processes. We found that paraneoplastic neuronal antigens 6A, a protein specific to humans, which shares 40–45% functional and structural homology with other paraneoplastic neuronal antigens family members [46,47], was significantly decreased in abundance in the hypothyroid state. The expression of these proteins is observed in systemic tumors, and they are considered factors that trigger an autoimmune response that subsequently leads to paraneoplastic neurological syndrome.

Network analyses using IPA demonstrated that the proteins identified in our dataset were involved in regulating IL-6 and TNF-α, which served as the central nodes with the highest connectivity in the pathway. IL-6 and TNF-α in turn cause the release of NF-κB, another important mediator of immune and inflammatory response through phosphorylation and degradation of inhibitor of κB. Clinical studies indicated that altered thyroid hormone concentration is associated with serum IL-6 concentration. IL-6, TNF-α, and NF-κB [48] are known to inhibit T3-dependent deiodinase enzyme activity, which further reduces the active TH concentration. These cytokines, known mainly to be produced from immune cells, are also secreted by the thyroid follicular cells, as well as by the inflammatory cells, are upregulated and increase the inflammatory reactions [49], leading to thyrocyte apoptosis and thyroid tissue damage. Administration of l-thyroxine reduces the cytokine levels to that of the euthyroid state. This finding is in line with those of Marchiori et al., who demonstrated an association between hypothyroidism and low-grade inflammation with significant reductions in pro-inflammatory cytokine (IL-1, IL-6, INF-γ, TNF-α) levels after l-thyroxine treatment [50]. 

## 4. Materials and Methods

### 4.1. Ethical Considerations and Informed Consent

All of the procedures and protocols were reviewed and approved by the Institutional Review Board at the College of Medicine, King Saud University (11 July 2010, registration number 10/2733/IRB). Written, informed consent was obtained from all the participants. This study was conducted at the Obesity Research Center, College of Medicine and King Khalid University Hospital (KKUH), King Saud University, Riyadh, Saudi Arabia. 

### 4.2. Study Design and Subjects 

Ten patients (6 females and 4 males, age: 39 ± 12.9 years) with newly diagnosed overt hypothyroidism referred to our endocrine outpatient clinic at KKUH were recruited prospectively for the 2D DIGE analysis. The N size was determined by carrying out a power analysis using the Progenesis same spots Nonlinear dynamics statistical software for the determination of minimum number of required biological replicate (Figure S1). Hypothyroidism was defined as a TSH level higher than 10 mIU/L and FT4 levels lower than 12 pmol/L. Samples were obtained from each patient at two time points: pretreatment samples (hypothyroid) were collected before starting l-thyroxine therapy and the post-treatment samples (euthyroid) were obtained from those with TSH level normalized after treatment with the appropriate dose of l-thyroxine for six weeks duration. If TSH levels were still above normal range, l-thyroxine dose was adjusted and the patients were seen after six weeks to confirm that TSH level is within normal range before obtaining the post-treatment samples. None of the patients that were recruited in the study had a history of hypertension, diabetes mellitus, inflammatory, or other autoimmune disorders. Blood samples were collected after a standard 10 h fast by venipuncture into EDTA-coated tubes and plasma was obtained by centrifugation (15 min, 3000× *g*), then were aliquoted and stored in multiple aliquots at −80 °C until analysis.

### 4.3. Biochemical Analysis

All of the parameters for biochemical and hormone analysis were carried out and determined using a Dimension Xpand Plus integrated clinical chemistry autoanalyzer (Siemens Healthcare Diagnostics, Deerfield, IL, USA). The serum levels of LDL cholesterol were calculated using Friedewald’s equation. Serum samples for twelve cytokines including TNF α and IL 6 was assayed using a customized Milliplex MAP Human Cytokine Panel HCYTOMAG-60K, (Merckmillipore, MA, USA) and the multiplex assay with the Luminex 200 Total System (Luminex Corp., Austin, TX, USA). Subjects’ serum samples were assayed according to the manufacturer’s protocols. Minimal detectable levels were as follows: IL-6 (0.053 pg/mL) and TNF α (0.031 pg/mL).

### 4.4. Sample Processing and Protein Extraction

Thawed plasma samples were centrifuged (5 min, 12,000× *g*) and depletion was carried out using an Albumin & IgG Depletion SpinTrap (GE Healthcare, Buckinghamshire, UK) for the high-abundance plasma proteins (albumin, IgG), according to the manufacturer’s instructions. Protein extraction was next carried out using TCA/acetone precipitation, as described by Chen et al. [51]. Briefly, the depleted plasma samples were mixed (1:4 ratio) with ice-cold acetone containing 10% *w*/*v* TCA and vortexed for 15 s for uniform mixing. The mixture was incubated at −20 °C for 2 h for protein precipitation. After incubation, tubes were centrifuged at 1000× *g* for 15 min at 4 °C, and the pellet was solubilized in labeling buffer (7 M urea, 2 M thiourea, 30 mM Tris-HCl, 4% CHAPS, pH 8.5). The protein concentration of each sample was next determined in triplicate using the 2D-Quantkit (GE Healthcare, Piscataway, NJ, USA). 

### 4.5. CyDye Labeling, 2-D DIGE, and Imaging

Equal amounts of protein (50 µg) from each sample, from both the hypothyroid and euthyroid groups were taken and labeled with 400 pmol of Cy3 and Cy5 dye. A mixture of an equal amount of all samples was pooled, labeled with Cy2, and used as an internal standard. A dye switching strategy was applied during labeling to avoid dye-specific bias (Table S1). First-dimension analytical gel electrophoresis was performed, followed by second-dimension sodium dodecyl sulfate polyacrylamide gel electrophoresis (SDS-PAGE) on 12.5% fixed gels, as previously described [52]. The 2D-DIGE gels were scanned and fluorescence images of the gels were acquired using a Typhoon 9400 variable mode imager (GE Healthcare, Uppsala, Sweden) at a 100 μM resolution employing the suitable excitation/emission wavelengths for each CyDye (Cy3 (523/580 nm), Cy5 (633/670 nm), and Cy2 (488/520 nm)) analyzed using the Progenesis Same Spots v.3.3 software (Nonlinear Dynamics Ltd., London, UK). Spot volumes were log transformed to generate normally distributed data and log normalized volume instead of spot intensities were used in statistical processing to quantify differential expression. All of the spots were pre-filtered and manually checked before applying the statistical criteria (ANOVA test, *p* ≤ 0.05 and fold ≥ 1.5). Independent direct comparisons were made between the protein spots relating to the hypothyroid and euthyroid groups and the fold differences and *p*-values were calculated using one-way ANOVA. Spots that fulfilled the above mentioned statistical criteria were submitted for further mass spectrometric (MS) analysis.

### 4.6. Colloidal Coomassie Blue Staining of the Preparative Gel

Total protein (1 mg) obtained from a pool of equal protein from the 20 plasma samples from both groups was separated by preparative two-dimensional (2D) gel electrophoresis. Gels were fixed in 40% (*v*/*v*) ethanol, 10% (*v*/*v*) acetic acid (overnight) and then washed (3×, 30 min each, ddH_2_O). The gels were incubated (1 h, 34% (*v*/*v*) methanol containing 17% (*w*/*v*) ammonium sulphate and 3% (*v*/*v*) phosphoric acid) prior to the addition of 0.5 g/L Coomassie G-250. After days, the stained gels were briefly rinsed with Milli-Q water and stored until the spots could be picked and identified by MS.

### 4.7. Protein Identification by MALDI-TOF MS

Coomassie-stained gel spots corresponding to the same spots that showed statistically significant differential abundance in the 2D-DIGE gels were excised manually and were washed and digested according to previously described methods [53]. The mixture of tryptic peptides (0.5 µL) derived from each protein was spotted onto a MALDI target (384 Anchorchip MTP 800 µm Anchorchip; Bruker Daltonik, Bremen, Germany) together with 0.5 μL of matrix (10 mg α-cyano-4-hydroxycinnamic acid (CHCA) in 1 μL of 30% CH_3_CN and 0.1% aqueous CF_3_COOH) and then left to dry (room temperature, RT) before MS analysis. Spectra were acquired on a MALDI-TOF MS (UltraFlexTrem, Bruker Daltonics, Bremen, Germany) in the positive mode (target voltage 25 kV, pulsed ion extraction voltage 20 kV). The reflector voltage was set to 21 kV and the detector voltage to 17 kV. Peptide mass fingerprints (PMF) were calibrated against a standard (peptide calibration standard II, Bruker Daltonics). The PMFs were processed using Flex Analysis software (version 2.4, Bruker Daltonics). MS data were interpreted by using BioTools v3.2 (Bruker Daltonics), together with the Mascot search algorithm (version 2.0.04 updated 9 May 2015; Matrix Science Ltd., London, UK). Mascot parameters were as follows: fixed cysteine modification with propionamide, variable modification due to methionine oxidation, one missed cleavage site (i.e., in case of incomplete trypsin hydrolysis), and a mass tolerance of 100 ppm. Identified proteins were accepted as correct if they showed a Mascot score greater than 56 and *p* < 0.05. Not all the spots of interest could be identified because some proteins were of low abundance and did not yield sufficiently intense mass fingerprints; other spots were mixtures of multiple proteins. 

### 4.8. Pathway Analysis 

Pathway analysis was carried out by importing quantitative data into the IPA software (Ingenuity Systems, http://www.ingenuity.com). This software aids in determining the functions and pathways that are most strongly associated with the protein list by overlaying the experimental expression data on networks constructed from published interactions.

### 4.9. Immunoblotting

To independently confirm the findings of the 2D-DIGE studies, proteins with significantly differential abundance were selected and examined by immunoblotting. Antibodies against C3 (SC-20137), haptoglobin (SC-69782), and α1 antichymotrypsin (SC-22747) were used. An equal amount of protein from each sample (50 µg) was separated by one-dimensional discontinuous slab gel electrophoresis (12% SDS-polyacrylamide gel). Proteins were electrotransferred to an Immobilon-P, polyvinylidene difluoride transfer membrane (PVDF, Millipore, Bedford, MA, USA), using a mini trans-blot electrotransfer cell (BioRad, Philadelphia, PA, USA). Following transfer, the membrane was stained with Ponceau-S to confirm transfer efficiency. The membrane was then blocked (5% fat free milk (FFM) in Tris-buffered saline (TBS), 1 h, RT) and rinsed (three washes of TBS-T in 10 mM Tris HCl, 150 mM NaCl, 0.1% Tween 20 buffer). Mebranes were then incubated with the appropriate primary antibody at its respective recommended dilution in blocking buffer, followed by incubation with the appropriate IgG-HRP-conjugated secondary antibody. The immunoreactive bands were detected by enhanced chemiluminescence (ECL, Thermo Scientific, Rockford, IL, USA), visualized by scanning with Fluorchem Q (Cell Biosciences, Santa Clara, CA, USA), and digitalized using the image analysis software Α view Q 3.0 (Cell Biosciences).

## 5. Conclusions

In summary, our proteomics analysis in the plasma of patients with hypothyroidism revealed distinct changes in expression of several acute-phase response proteins from the classical response. The involvement of these proteins in the pathophysiology of thyroid disease is in conceptual agreement with previous biochemical, molecular, and proteomic studies. The normalization or near-normalization of these proteins after treatment would add to our understanding of the clinical and biochemical changes we see after treating patients with hypothyroidism. Further mechanistic studies should be undertaken to understand the clinical implications of the changes in these proteins.

## Figures and Tables

**Table 1:** Biochemical parameters of the study subjects at baseline and after l-thyroxine therapy.

Parameters	Hypothyroid	Euthyroid	*p* Value
Number of patients	10	10	
Fasting glucose (mmol/L)	5.3 ± 0.4	5.0 ± 0.5	0.078
Urea (mmol/L)	4.7 ± 0.7	4.6 ± 0.9	0.392
Creatinine (mmol/L)	72.5 ± 13.1	76.1 ± 23.3	0.404
Aspartate transaminase (IU/L)	33.4 ± 6.6	37.5 ± 9.0	0.407
Alanine transaminase (IU/L)	18.0 ± 5.8	17.4 ± 3.9	0.169
Alkaline phosphatase (IU/L)	94.9 ± 25.9	96.8 ± 31.2	0.133
Haemoglobin (g/L)	13.1 ± 1.7	13.5 ± 2	0.50
FT4 (pmol/L)	8.3 ± 5.5	18.8 ± 3.7	0.000
TSH (mIU/l)	33.9 ± 22.1	1.6 ± 0.9	0.000
Total Cholesterol (mmol/L)	4.6 ± 0.6	4.8 ± 0.7	0.193
Triglycerides (mmol/L)	1.2 ± 0.3	1.4 ± 0.3	0.184
High Density Lipoprotein cholesterol (mmol/L)	2.9 ± 0.8	3.0 ± 0.6	0.373
Low density Lipoprotein cholesterol (mmol/L)	1.2 ± 0.4	1.0 ± 0.3	0.087
TNF-α	4.7 ± 1.4	3.2 ± 2	0.095
IL-6	1.0 ± 0.65	0.68 ± 0.35	0.087

**Table 2:** List of significantly differentially expressed proteins identified in human plasma between hypothyroid vs. euthyroid states using 2D-DIGE. Differences in fold change are shown. Protein name, accesion number, Mascot score, MS% coverage, protein MW, and pI values according to Uniprot database are listed.

Accession No ^a^	MASCOT ID	Protein Name	Pi ^b^	MW ^c^	Coverage %	Score ^d^	Ratio of Hypothyroid/Euthyroid	Fold Change ^e^	*p*-Value
P06681	CO2_HUMAN	Complement C2	7.23	83,268	22	64	up	3.3	0.002
P02787	TRFE_HUMAN	Serotransferrin	6.81	77,000	45	67	up	2.2	0.002
P01024	CO3_HUMAN	Complement C3	6.39	187,030	14	60	up	1.7	0.006
P01834	IGKC_HUMAN	Ig κ chain C region	6.11	11,602	85	57	up	2.2	0.009
P01011	AACT_HUMAN	α-1-antichymotrypsin	5.33	47,621	27	72	up	2.3	0.009
P0C0L4	CO4A_HUMAN	Complement C4-A	6.66	1,926,500	28	63	up	3.4	0.01
P00738	HPT_HUMAN	Haptoglobin	6.3	45,177	68	60	up	1.9	0.015
P02671	FIBA_HUMAN	Fibrinogen α chain	5.10	94,914	23	75	up	1.7	0.010
P02647	APOA1_HUMAN	Apolipoprotein A-I	5.56	30,759	34	69	up	2.2	0.013
P01876	IgGA1_HUMAN	Ig α-1 chain C region	6.08	37,631	45	166	up	1.5	0.042
P08603	CFAH_HUMAN	Complement factor H	6.23	139,068	27	72	down	2.5	0.033
P0CW24	PNM6A_HUMAN	Paraneoplastic antigen-like protein 6A	5.24	43,875	35	133	down	2.1	0.034
P01023	A2MG_HUMAN	α-2-macroglobulin	6.00	163,189	27	69	down	1.9	0.035

MW-molecular weight; ^a^ Protein accession number for SWISSPROT Database; ^b^ Theoretical isoelectric point; ^c^ Theoretical relative mass; ^d^ MASCOT score; ^e^ Protein expression between hypothyroid and euthyroid states.

## Supplementary Materials

Supplementary materials can be found at www.mdpi.com/1422-0067/19/1/88/s1.
