# Supplementary material for: Differences in the Plasma Proteome of Patients with Hypothyroidism before and after Thyroid Hormone Replacement: A Proteomic Analysis
Source: Int J Mol Sci. 2018 Jan 1;19(1):88. doi: 10.3390/ijms19010088 (PMC5796038; doi:10.3390/ijms19010088)

Supplementary

Table S1. Experimental design: 20 patient samples run on 10 2D-PAGE gels; samples were labeled randomly with Cy3 and Cy5, and a pooled sample stained with Cy2 was used as an internal standard.

Gel	Cy3	Cy5	Cy2
1	1 (Euthyroid)	11 (Hypothyroid)	Pooled sample
2	12 (Hypothyroid)	2 (Euthyroid)	Pooled sample
3	3 (Euthyroid)	13 (Hypothyroid)	Pooled sample
4	14 (Hypothyroid)	4 (Euthyroid)	Pooled sample
5	5 (Hypothyroid)	15 (Euthyroid)	Pooled sample
6	16 (Euthyroid)	6 (Hypothyroid)	Pooled sample
7	7 (Hypothyroid)	17 (Euthyroid)	Pooled sample
8	18 (Euthyroid)	8 (Hypothyroid)	Pooled sample
9	9 (Hypothyroid)	19 (Euthyroid)	Pooled sample
10	20 (Hypothyroid)	10 (Euthyroid)	Pooled sample

Table S2. Canonical pathways and network Pathways obtained from IPA functional analysis.

INGENUITY
PATHWAY ANALYSIS

Analysis Name: Hypothyroid for IPA - 2016-10-27 12:08 PM
Analysis Creation Date: 2016-10-27
Build version: 400896M
Content version: 28820210 (Release Date: 2016-09-24)

Analysis Settings

Reference set: Ingenuity Knowledge Base (Genes Only)
Relationship to include: Direct and Indirect
Does not Include Endogenous Chemicals
Optional Analyses: My Pathways My List

Top Networks

ID	Associated Network Functions	Score
1	Neurological Disease, Immunological Disease, Metabolic Disease	28
2	Cell-To-Cell Signaling and Interaction, Cellular Compromise, Cellular Function and Maintenance	3

Top Canonical Pathways

Name	p-value	Overlap	
Acute Phase Response Signaling	2.35E-17	5.3 %	9/169
Complement System	3.91E-09	10.8 %	4/37
LXR/RXR Activation	4.36E-09	4.1 %	5/121
FXR/RXR Activation	5.35E-09	4.0 %	5/126
Coagulation System	1.74E-04	5.7 %	2/35

FigureS1: Power calculation for determination of the minimum number of required biological variants for 2-DIGE analysis. The power curve was used to calculate the sample size required to find significant difference with a fold-change of ≥1.5 between two paired groups at 80% power and p-value ≤ 0.05.

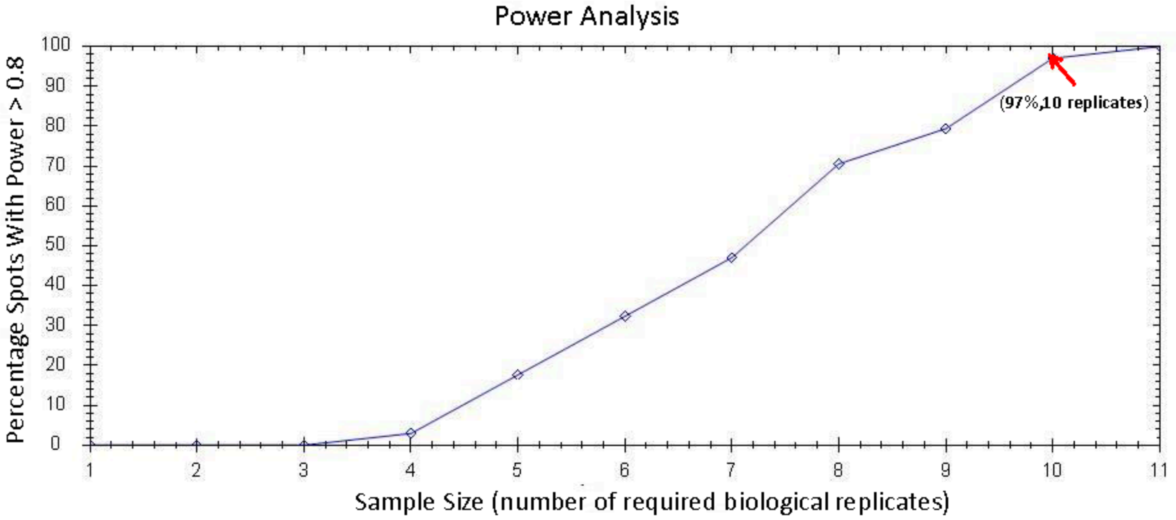

Figure S2: Representative focussing of the 13 protein spots between the euthyroid and hypothyroid samples on the 2D-DIGE gel images and their expression between the two groups.

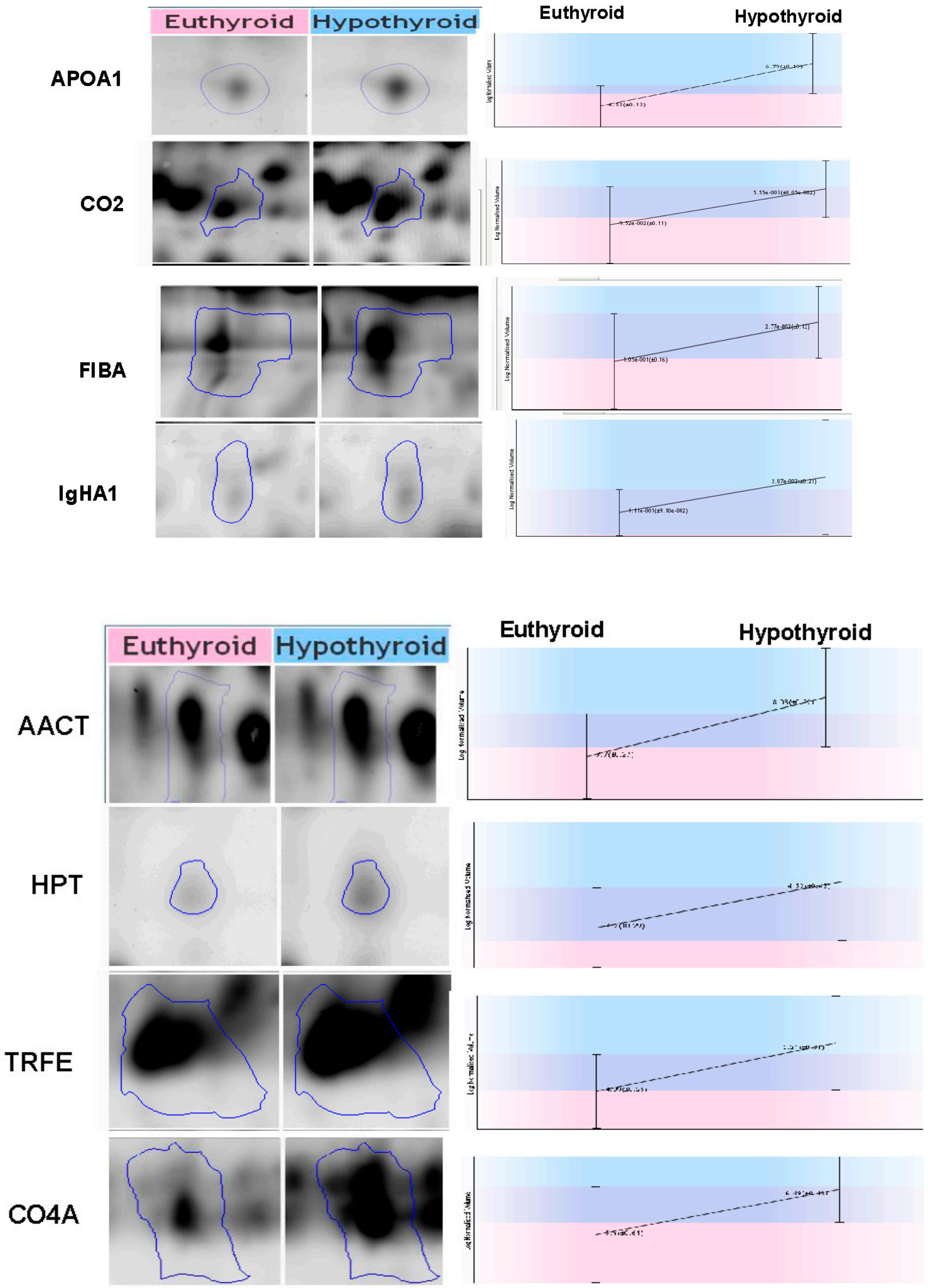

Supplement: Supplementary file 1 [file ijms-19-00088-s001.pdf]
